# Mixed Random and Nonrandom Allocation, and Group Randomization Have Been Mislabeled and Misanalysed, Necessitating Reanalysis. Comment on Conner et al. KiwiC for Vitality: Results of a Randomized Placebo-Controlled Trial Testing the Effects of Kiwifruit or Vitamin C Tablets on Vitality in Adults with Low Vitamin C Levels. *Nutrients* 2020, *12*, 2898

**DOI:** 10.3390/nu14194062

**Published:** 2022-09-30

**Authors:** Colby J. Vorland, Yasaman Jamshidi-Naeini, Lilian Golzarri-Arroyo, Andrew W. Brown, David B. Allison

**Affiliations:** 1 Department of Applied Health Science, Indiana University School of Public Health-Bloomington, Bloomington, IN 47405, USA; 2 Department of Epidemiology and Biostatistics, Indiana University School of Public Health-Bloomington, Bloomington, IN 47405, USA

We read the report by Conner and colleagues that tested whether kiwifruit or vitamin C affected measures of vitality [1]. The trial is reported as a randomized controlled trial (RCT), but not all participants were allocated randomly, and some participants were group-randomized, which was not accounted for in the analysis. Together, these design choices mean that as published, the statistical analysis and interpretation of results as an RCT are not warranted and corrections are needed.

We contacted the authors to inquire about their design after reading their methods, which included references to ‘batch randomization’ and challenges with stratification. The authors expeditiously shared their data, code, and additional detail on their design. We thank the authors for their collegiality in communication.

Through discussion, we learned that some participants were not randomly assigned, while some were group-randomized, and others individually randomized (see figure at https://doi.org/10.31219/osf.io/ehk3x (accessed on 26 April 2021) for our understanding of the design). We elaborate on the different aspects of nonrandom, random, restricted random, and group random methods at https://doi.org/10.31219/osf.io/ehk3x (accessed on 26 April 2021). The design choices change requirements for the analysis and communication of the trial as an RCT. We outline two errors below.

First, the trial should not be labeled as an RCT because of the methods used. Randomization is generally considered the gold standard to provide unbiased estimates of causal effects [2]. The use of some nonrandom methods, such as assigning kiwifruit to the first groups of participants because of availability, as done by Conner et al., does not permit the causal inferences justified by randomization [3,4], and therefore makes it incorrect and inappropriate for the KiwiC study to be called an RCT.

Second, when participants are randomized in sets of two or more participants (as opposed to each participant being randomized individually), this is conventionally referred to as group-randomized or cluster-randomized and referred to by Conner et al. as ‘batch randomized’. With group-randomization, the model residuals are ‘correlated’ (i.e., the residuals are not independently and identically distributed, *iid* [5]) and this violation of the assumption of standard analyses must be taken into account during the analysis [6], but they were not. As stated by Murray and colleagues: “Any test that ignores either the extra variation or the limited degrees of freedom will have a type I error rate that is inflated…” [7].

We encourage the authors to provide readers additional detail on their methods as they generously shared with us, which is essential for interpreting this study. We note a similar example of the Primary Prevention of Cardiovascular Disease with a Mediterranean Diet (PREDIMED) trial, in which, just as in this trial, the nonrandom allocation of some of the participants and some group-randomizations were uncovered in a trial audit [8]. In that case, the paper was retracted and republished with a reanalysis to properly consider these factors [8]. The ‘retract and republish’ approach allows for critical issues to be corrected and clarified while simultaneously acknowledging the value of a study through republication. Until corrections are made, causal inferences from this paper are not warranted.
